# Sprint Performance Changes and Determinants in Afro-Caribbean Adolescents Between 13 and 15 Years Old

**DOI:** 10.2478/v10078-012-0067-8

**Published:** 2012-10-23

**Authors:** Karine Babel Copaver, Claude Hertogh, Olivier Hue

**Affiliations:** 1 Laboratory A.C.T.E.S. UPRES-EA 3596, U.F.R.S.T.A.P.S. – U.A.G.Campus de Fouillole, Pointe-à-Pitre Cedex, France.

**Keywords:** puberty, stride characteristics, sprinting, Afro-Caribbean youth

## Abstract

Afro-Caribbean sprinters often reach high performance levels at an early age. Adolescence is a time of morphological and physiological changes. This study was designed to analyze the evolution in parameters of short sprint performance during adolescence in Afro-Caribbean boys, especially the stride number/body height ratio (SN/BH), which is at the interface of technical and morphological factors. Seventy-one 13-year-old boys performed vertical jumps and short sprint races. The races were filmed with a view to determine stride variables. Anthropometric parameters were also measured. The same tests were performed two years later. Body height and SN/BH were the main predictors of sprint performance. The delta of performance was principally explained by stride length and stride number. Although deterioration in technical parameters was expected, the parameters related to body size and stride length were the main sprint performance predictors rather than explosive force. These results could be useful in developing tests to detect sprint potential in youth.

## Introduction

African-Americans and West Indian athletes have long dominated international sprint events and this was evident in the 2009 and 2011 World Track and Field Championships. A few studies have focused on the influence of ethnicity on sprint performances. Most of them demonstrated that black boys and girls performed better than their white counterparts of the same age in 30 to 50 m dashes (Milne et al., 1976), although others showed no differences (Babel et al., 2005). Concerning physiological factors, although some studies demonstrated that bone density was higher (Schutte, 1984; Wang et al., 1999) and fat content lower in blacks (Himes, 1988), it seems that muscular architecture is not influenced by ethnic origin (Abe et al., 1999). Still other studies have demonstrated a more advanced puberty for black boys than white (Morisson et al., 2000). In sprint running, several elite Afro-Caribbean athletes have performed exceptionally well at an early age (Usain Bolt, the fastest man in the world, had already run the 200 m in 21.73 s in 2001 at the age of 14). In 2005, Babel et al. demonstrated that ethnicity did not influence sprint performance in prepubertal boys, but the predictive variables of performance were better for Afro-Caribbean boys versus Caucasians. We thus wondered how sprint performance and its variables changed in Afro-Caribbean boys during adolescence.

Children’s physical resources are transformed in both qualitative and quantitative ways during development, and physical performance varies with age and sex (Weineck, 1996). During the first pubertal stage (from 12–13 to 14–15 years for boys), the abrupt body changes and hormonal instability cause a decline in specific coordination (Weineck, 1996), a decrease in the precision of body control, and excessive movement. The release of specific sexual hormones also causes significant morphological changes (Thiebaud, 1997).

In addition to morphological factors, muscle strength also undergoes change during puberty (Round, 1999; Jones et al., 2004), what affects physical performance. Indeed, pubertal development and muscle strength are closely related. Malina et al. (2004) tested 69 young football players (13 and 15 years old) and showed that both biological maturity (stages of puberty) and training significantly influenced their functional capacities. Moreover, their study indicated that, although training contributed to an increase in aerobic capacity, sprint and jump performances were more related to anthropometric variables (notably body mass and body size). Chandler and Brown (2007) showed that improvement in sprint performance was due to an increase in stride frequency followed by an increase in stride length. The morphological changes induced by development and maturation most likely influenced sprint performance.

In 2005, Babel et al. demonstrated that the stride number/body height ratio (SN/BH) could be used to predict sprint performance in Afro-Caribbean children. This ratio is interesting because it includes both technical (stride frequency and length) and anthropometric variables. The study showed that the fastest runners were those with the lowest SN/BH ratio, meaning that those with the smallest number of strides in relative values had the best results in short sprint performance. We thus raised the question of how maturation would influence this last variable.

We were specifically interested in how the SN/BH ratio would change so we decided to test its persistence as a predictive variable of sprint performance during adolescence. Great changes in anthropometric variables occur during adolescence, notably an increase in body size. These changes can lead to instability in coordination (Weineck, 1996) and might reduce the importance of the SN/BH ratio as a predictive variable of sprint performance. It can thus be hypothesized that changes in anthropometric variables will perturb coordination and have a negative influence on sprint performance. Greater maximal leg strength has been demonstrated to be strongly correlated with sprint performance (Kumagai et al., 2000; Bret et al., 2002). The literature shows that leg strength is correlated with jump tests, particularly vertical jump height (Sleivert and Taingahue, 2004). As strength increases during puberty and is closely related to sprint performance, we assumed that vertical jump height would show greater predictive power regarding sprint performance. We also assumed that over the course of adolescence, sprint performance would be greater due to the increase in leg strength. We therefore tested the hypothesis that between 13 and 15 years, vertical jump height would become a better predictor of sprint performance than the SN/BH ratio.

## Material and Methods

### Subjects

Two hundred youths participated in the first part of this study. At least 70 performed all the tests in Years 1 and 3. Subjects were lost to analysis for many reasons, mainly school changes, involvement in sports competition, or injuries. The average age of the subjects at the start of the study was 13.24 ±1.05 years. The ethics committee of the University of the French West Indies approved the study, and parental consent was obtained for all subjects who participated in the study. All measurements and tests were conducted within the confines of the school, during physical education classes. Authorization was first obtained from the headmaster.

All tests were conducted twice, two years apart. During the interval, none of the subjects was involved in athletic practice and/or competition. The only physical activities were the official physical education classes for up to 3 hours per week, depending on the class. The conditions for the testing procedures and the equipment were the same in the two testing periods.

### Anthropometric variables and puberty ratings

Body height and leg length were measured with a wall meter and body mass was measured with a calibrated scale. Leg length was measured in a lateral position, from the anterior superior spine of the ilium to foot contact with the ground (Mak et al., 2006). The dominant leg was measured. For right-handed individuals, the dominant leg is generally the left one, but we considered the leg spontaneously used by subjects to jump as dominant. The subjects stood with the feet placed shoulder-width apart. They were asked to stand straight and look straight ahead. The percentage of body fat was obtained from skinfold thickness measured at four sites (biceps, triceps, supra iliac and subscapular) with a caliper (Caliper Holtain Ltd, Crymyych, UK), as described by Durnin and Rahaman (1967).

Sexual maturation was evaluated by the pubertal stages of Tanner (1989). To determine the stage, five illustrations were shown to the children as described by Taylor et al. (2001).

### Physical tests

The subjects took part in a standardized protocol consisting of a vertical jump test and a 30 m sprint test.

#### Jump test

The jump test was performed using Abalakov aparatus (Jump-MD, Takei, Japan). Performance was assessed by the unwinding of a thin cord tethered at the waist. The amount of cord unwound automatically appeared on a digital screen fixed to the belt. The jump height was given with a precision of 1 cm. The participants were asked to perform a countermovement jump in which they began in a standing position, dropped into a semi-squat position, and immediately jumped as high as possible. Knee flexion was monitored by an experimenter in order to prevent excessive or insufficient flexion. The jumps were performed without the help of the arms, which remained on the hips. Each subject performed three jumps separated by 4 minutes of rest to allow for ATP/PC resynthesis. The vertical jump measure retained was the best of the three jumps.

#### Sprint performance

The sprint tests were performed individually in one lane of a synthetic track. The distance was delimited by two markers at the start line and two others at the finish line. Before beginning the test, the subjects warmed up for about 20 minutes with a slow run, dynamic stretching and some specific exercises. The subjects performed the same warm-up at both testing periods. Every 30-m sprint was filmed with a digital video camera (VL-WD250S, Sharp, Malaysia; speed, 1/1000 to 1/10000 s) positioned at a 14° angle to the sprint start. The camera was located about 20 m from the start line with a lateral distance of 5 m from the lane. The film analysis consisted of counting the number of strides during the sprints using the camera view-by-view function. A stride was defined as a bound between the right and left contact with the ground and was identified at each foot contact with the ground. The performance time was assessed using photoelectric cells (Globus Tecnica e Sport, Italy) placed at the start and finish lines. The subject stood 1 m behind the line. When he was ready, he began to run. All children were vocally encouraged during the sprints. Each participant performed only one sprint. All wore their usual sports shoes. Cells were placed at 1 m in year 1, then at 110 cm in year 3.

### Statistics

Paired Student tests were conducted to determine the differences between the performances during the two years of research. To determine the variables predictive of sprint performance, multiple correlations were also performed on the overall performance and the performance deltas between the two years.

## Results

### Anthropometric and performance differences between Year 1 and Year 3

The age of participants increased from 13.24 to 15.24 years. The anthropometric parameters showed that body height and mass had increased significantly by year 3, respectively 157 versus 170 cm (p < 0.01) and 47 versus 64 kg (p < 0.01). There was no significant difference in fat content (19.85 versus 18.29 %).

The physical fitness tests showed a significant improvement in running speed and vertical jump height. Stride frequency was the only variable that did not change. Stride length increased significantly, and the SN/BH ratio showed a significant decline.

### Predicting sprint performance

A system of stepwise regressions revealed four predictors of performance in the following equation:

$$\begin{array}{c} \text{Perf}=0.016\;\text{BH}+22.086\;\text{SN}/\text{BH}-1.236\;\text{SF}-1.451\;\text{SL}+6.817\\ {\text{r}}^{2}=0.995,\;\text{p}=0.001 \end{array}$$

BH: body height, SN/BH: stride number/body height ratio; SF: stride frequency; SL: stride length

### Variables related to sprint performance

Table 1 shows the correlations between the measured variables and sprint performance. All the parameters were correlated with the 30-m sprint performance.

### Variables correlated with sprint performance

Except for body mass, the changes in all parameters were significantly, but weakly correlated with performance.

### Predictive parameters of the difference in performance

No anthropometric variable emerged as predictive in the equation from the regression step. The performance predictors were stride length, the number of strides, and stride frequency.

$$\begin{array}{c} \text{Delta}\;\text{Perf}=-0.453\;\text{SL}+0.217\;\text{SN}30-1.252\;\text{F}-0.009.\\ {\text{r}}^{2}=0.989,\;\text{p}=0.001 \end{array}$$

SL: stride length, SN: stride number, SF: stride frequency

Stride frequency did not change over the two years, and it remained one of the predictive variables of performance.

## Discussion

The most important results of this study were as follows: 1) the lack of correlation when simple regressions were used, whereas 2) step-by step regression revealed a change in the determinants of performance and confirmed the importance of stride length in sprint running.

### Changes in anthropometric, physical and technical variables

Afro-Caribbean boys distinguish themselves in sprint track running even at an early age. The literature is controversial concerning the influence of ethnic origin on sprint performance. To our knowledge, no study has demonstrated that the factors that determine performance differ with ethnic origin. In 2005, Babel et al. showed that the factors of performance were the same for Afro-Caribbean and Caucasian prepuberatl boys (vertical jump and stride number/height ratio). However, they also showed that Afro-Caribbeans had better results concerning these factors.

The vertical jump significantly improved over the two-year period. We did not evaluate hormonal concentrations and can only assume that this improvement was due to the rise in testosterone production, which influences muscle growth, strength and vertical jump height (Cardinale and Stone, 2006). Moreover, age rather than pubertal stage seems to be correlated with a testosterone level (Srinivasan, 1986). The literature is controversial in regard to the influence of ethnicity on testosterone and growth hormones. Some authors showed that prepubertal and young black boys had higher testosterone levels (Ross et al., 1986; Winters et al., 2001; Abdelrahamane et al., 2005) and higher insulin growth factor (IGF-1) (Higgins et al., 2005). However, other authors demonstrated that ethnicity did not have any effect on a testosterone level during childhood and puberty (Lee et al., 2010; Morisson et al., 2000; Richards et al., 1992).

The results of the present study are consistent with the literature on the development of motor skills: boys and girls improve during the first 18 years (Costill and Wilmore, 2006), although the improvement in physical fitness is not linear during development (Philipaerts et al., 2006). Body fat content was the only anthropometric variable that did not vary significantly. We observed only a trend (p < 0.06), whereas a significant reduction based on earlier findings of a 4% decrease in body fat between the age of 13 and 15 was expected (Wabtish, 1997). In addition to the improved sprint performance and the increase in anthropometric indices, stride length increased and the stride number and SN/BH ratio decreased, what was probably related to the increase in body size. Stride frequency apparently stabilized as it showed little difference between the initial and final evaluations. Concerning the stride frequency/length factor, the speed improvement was mainly due to longer strides. These results are therefore consistent with the literature (Chandler and Brown, 2007)

### The determinants of performance

The main predictive variables of performance were body height and the SN/BH ratio, underlining the importance of anthropometric characteristics. The literature nevertheless shows controversial findings. Some authors showed that changes in some of the anthropometric parameters (body size, body mass) improved physical performance (Van Praagh and Dore, 2002). Other studies reported that the standard anthropometric factors were low performance predictors (Kukolj et al., 1999), and low stride length predictors for distance running (Cavanagh and Williams, 1982).

The finding that body size was the main predictor of performance may suggest that the taller an individual is, the longer his legs are likely to be and thus the longer the strides will be, as well. Usain Bolt (1.96 m), the current world record holder for the 100 m (9.58 s), illustrates these characteristics, although tallness is not characteristic of all elite sprinters (Greene 1.75 m, 9.79 s; Nesta Carter 1.78 m, 9.78 s; Ato Boldon 1.75 m, 9.86 s; Tyson Gay 1.83 m, 9.69 s).

One of our objectives was to test the SN/BH ratio. The results of the stepwise regression confirmed its relevance in predicting sprint performance as it was the second most important predictive parameter, after body size. This parameter shows the adaptation of stride length to a morphological variable, body size, during sprint racing. This interaction is necessary and indicates a feedback relationship and not simply a one-way relationship. Thus, the SN/BH ratio can be used as an index of coordination and efficiency specific to sprint racing in children and adolescents.

### Determinants of change in performance

Regression analysis was performed on the delta of performance between the first and third year of the experiment. The results showed that sprint performance changed significantly, because stride length increased, the stride number slightly decreased, and the stride rate did not change. It thus seems that the variables related to stride length determine the change in sprint performance during adolescence. In the fastest sprinters, stride length increased while a stride number decreased.

Stride length is an important performance variable in sprint races of elite athletes (Summers, 1997). The results of the present study showed that this variable also determines performance in the sprints of sedentary children and adolescents, indicating that the running technique of elite sprinters (Morin et al., 2003) optimizes the same variables that determine sprint performance in non-athletes.

Of course, elite performance includes many factors that cannot be summarized in one or two variables. In 1996, Krantz studied and compared the stride patterns of Michael Johnson (MJ) and Marie-Jose Perec (MJP) at the 1996 Atlanta Olympic Games. Whereas MJ had a racing pattern showing more grasp than push, MJP showed greater push than traction. This difference may be explained by the specificity of their anthropometric features and the need to adapt their stride to achieve cost-effective performance. Despite their differences in racing pattern, both have been the best in their respective disciplines.

The results of this study show that performance improved with the increase in stride length. Many authors have demonstrated the relationships between stride length, sprint performance and ground contact time (Derrick et al., 1998; Mercer et al., 2002; Coh et al., 2001). Ground contact time was not measured in our study. However it raises the question of the relationship between strength and coordination. Indeed, better ground support could be interpreted as a better use of lower limb strength or simply as the result of stronger lower limbs.

Many authors have shown the relationship between physical fitness test results and pubertal stages. Pineau (1991) demonstrated that during puberty, explosive strength and vertical jump height changed in parallel with statural characteristics between Tanner stages 1 and 3. Bucheit (2008) demonstrated a significant correlation between short sprint test results and pubertal stage, from stage 2. In sprint running, since technical factors are so important, the role of coordination also needs to be addressed. Stride frequency and stride length must be in correct proportions to be efficient (Mero et al., 1992), but the variables representing stride length are widely used in predicting performance in sprint racing. Stride length reflects power, coordination, and the adaptation to morphological constraints. From 12 to 15 years for boys, the abrupt body changes and hormonal instability lead to a decrease in specific coordination (Cardinale and Stone, 2006). In the present study, we expected that disturbances in coordination would affect the stride pattern, with stride frequency and amplitude becoming unstable performance variables. This was not the case. Thus, our results indicated that the sprint coordination patterns were not affected by the strong morphological changes associated with maturation. Indeed, it seems that not all types of motor function are affected by changes in body proportion (Weineck, 1996). In the present study, the increases in body size and muscle strength probably contributed more to increasing stride length, rather than affecting the race pattern.

Contrary to our hypothesis, the stepwise regression did not identify vertical jump height as a predictive variable of performance. According to findings on the relationship between lower limb strength and sprint performance (Morin and Belli, 2003; Sleivert and Taingahue, 2004), we can assume that the change in explosive power of the lower limbs would match changes in sprint running performance. One explanation is that during adolescence, the ability to run fast is not simply related to great ground forces, but also to a better impact of strength on stride length. Thus, better results on strength tests do not inevitably lead to greater sprint performance, which probably explains why strength tests are not systematically correlated with running velocity (Morin and Belli, 2003).

In conclusion, this study shows that although vertical jump height is most often described as the strongest predictor of sprint performance with increasing maturity, we found that it remained strongly correlated with sprint performance, but less so than stride length, which was a predictive variable for adolescents between 12 and 15 years old. Furthermore, it appears that with greater maturity, the subjects of this study spontaneously adopted a style of running similar to the current techniques of sprint racing. The stride number/height ratio seems to be a valid predictor of sprint performance, but this finding should be confirmed up to the last stage of puberty. In order to detect sprint potential, coaches should use a larger battery of tests that includes the assessment of stride length, which should be put into relation with body size. Further research is needed to confirm these results up to the last stage of puberty and to study the variables of stride length, particularly ground contact during sprinting.

## Figures and Tables

**Figure 1:**
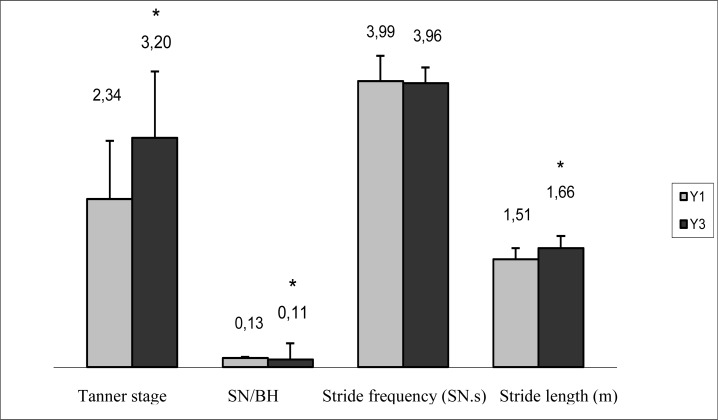
Changes in pubertal stage, SN/BH ratio, and stride characteristics. SN/BH: stride number/body height ratio. Stride frequency in stride number per second (SN.s). Stride length in meters (m). Y: Year. *p<.001

**Figure 2:**
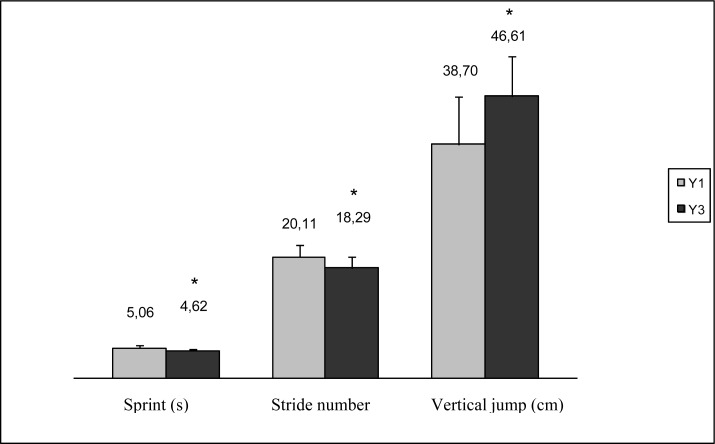
Changes in sprint performance, stride number and vertical jump height. *p<.001. Sprint in seconds (s), vertical jump in centimetres (cm)

**Table 1:** Relationships between sprint performance (30 m) and anthropometric variables as well as chosen fitness tests

	**Age**	**Tan**	**BH**	**BM**	**FM %**	**SN**	**VJ**	**SN/BH**	**SF**	**SL**
	
**r^2^**	.255	.575	.385	.030	.221	.561	.561	.643	.031	.565
**p**	<.001	<.001	<.001	<.05	<.001	<.001	<.001	<.001	<.05	<.001

Tan: Tanner stage, BH: body height, BM: body mass, FM%: fat mass percentage, SN: stride number, VJ: vertical jump, SN/BH: stride number/body height ratio, SF: stride frequency, SL: stride length

**Table 2:** Relationships between the deltas in sprint performance of boys aged 11 – 13 and anthropometric variables as well as chosen fitness tests

	**BH**	**BM**	**SN**	**FM %**	**VJ**	**SN/BH**	**SF**	**Stride length**
	
**r^2^**	.073	.031	.209	.074	.114	.266	.054	.197
**p**	<.05	.144	<.001	<.05	<.01	<.001	<.05	<.001

BH: body height, BM: body mass, SN: stride number, FM%: fat mass percentage, VJ: vertical jump, SN/BH: stride number/body height ratio, SF: stride frequency.
